# Association between waist circumference and chronic pain: insights from observational study and two-sample Mendelian randomization

**DOI:** 10.3389/fnut.2024.1415208

**Published:** 2024-07-26

**Authors:** Ting Xu, Fan Jin, Yeting Yu, Jie He, Ren Yang, Tian Lv, Zhangjun Yan

**Affiliations:** ^1^Department of Anesthesiology, Traditional Chinese Medical Hospital of Zhuji, Zhuji, China; ^2^Department of Anesthesiology, Zhuji People's Hospital, Shaoxing University, Zhuji, China; ^3^Department of Neurology, Zhuji People's Hospital, Shaoxing University, Zhuji, China

**Keywords:** waist circumference, chronic pain, NHANES, Mendelian randomization, inverse variance weighted

## Abstract

**Background:**

Current research offers limited clarity on the correlation between waist circumference and chronic pain prevalence.

**Objective:**

This investigation seeks to elucidate the potential relationship between waist circumference and chronic pain and their causal association.

**Methods:**

An observational study was conducted, leveraging data from the National Health and Nutrition Examination Survey (NHANES) collected between 2001 and 2004. The multivariable logistic regression was used to assess the relationship between waist circumference and chronic pain. Furthermore, a meta-analysis of Mendelian Randomization (MR) was applied to explore a causal relationship between waist circumference and pain.

**Results:**

The observational study, post multivariable adjustment, indicated that an increase in waist circumference by 1 dm (decimeter) correlates with a 14% elevation in chronic pain risk (Odds Ratio [OR] = 1.14, 95% Confidence Interval [CI]: 1.04–1.24, *p* = 0.01). Moreover, the meta-analysis of MR demonstrated that an increased waist circumference was associated with a genetic predisposition to pain risk (OR = 1.14, 95%CI: 1.06–1.23, *p* = 0.0007).

**Conclusion:**

Observational analysis confirmed a significant relationship between increased waist circumference and the incidence of chronic pain, and results based on MR Study identified increased waist circumference as potentially causal for pain.

## 1 Introduction

Chronic pain, now recognized as a substantial global health issue, afflicted nearly one-third of the world’s population, as indicated in recent literature (1). In the United States, 2016 statistics showed that approximately 20% of adults, equating to nearly 50 million individuals, were afflicted by chronic pain, with 8% experiencing severe forms of this condition (2). Similarly, in the United Kingdom, a significant number of people suffer from chronic pain, with estimates ranging from one-third to half of all adults, approximating nearly 28 million individuals. The challenge was expected to escalate with the demographic shift towards an older population (3). Conditions such as lower back pain and migraines, significantly contributed to the burden of disability and illness, were notably documented in the Global Burden of Disease Study 2016. Even in nations with lower prevalence rates, like China, chronic pain affected a substantial number of individuals, estimated at 9,201 per 100,000 people (4).

Concurrently, obesity presents as another critical public health concern worldwide. World Health Organization data from 2016 indicated that 39% of adults aged 18 and above were overweight, with a concerning 13% classified as obesity (5). Research demonstrated a strong positive association between obesity and chronic pain (6–9). However, these studies predominantly utilized Body Mass Index (BMI) as the measure for obesity, a method with notable limitations in differentiating fat-free mass (FFM) from fat mass (FM) and in providing insights into fat distribution (10). Recent studies suggested that BMI alone may be insufficient for clinicians to accurately evaluate and address health risks associated with obesity (11).

Waist circumference, a straightforward and clinically practical measure, was recommended by several health institutions for assessing central obesity (12). It was considered a better measure of visceral fat than BMI (13, 14), more sensitive in identifying Metabolic Syndrome (MetS) (15, 16), and offered improved assessment of health outcomes in older adults compared to BMI (11). In addition, Beverley Balkau (17) verified that waist circumference was more strongly linked to the incidence of heart diseases and diabetes than BMI. Recent research has linked waist circumference with all-cause mortality (18), cardiovascular mortality (19, 20), Type 2 diabetes (21), and cognitive decline in older adults (22). The correlation between waist circumference and chronic pain, akin to that of BMI, remains a topic of ongoing debate and warrants further investigation.

Therefore, this study utilized cross-sectional data from the National Health and Nutrition Examination Survey (NHANES), a comprehensive and reliable source of health information in the United States. By integrating Mendelian randomization (MR) causal analysis, a method that employs genetic variants as instruments to infer causality, the study aimed to provide a clearer understanding of the potential causal link between waist circumference and the experience of chronic pain, accounting for both genetic and environmental factors.

## 2 Methods

### 2.1 NHANES

#### 2.1.1 Study sample and design

The NHANES began in the 1960s, which was designed to evaluate the health and dietary conditions of individuals in the United States. The results played a crucial role in establishing the frequency of significant illnesses and potential risks, as well as assessing the nutritional condition for the purpose of promoting health and preventing diseases (23). The NHANES data, an important cornerstone of nutritional surveillance in the United States (24), includes comprehensive questionnaire data on chronic pain from over 7,000 individuals, with 1,129 reporting chronic pain, alongside relevant metrics such as waist circumference and BMI. This dataset forms a robust foundation for exploring the cross-sectional relationship between waist circumference and chronic pain.

**Figure 1:**
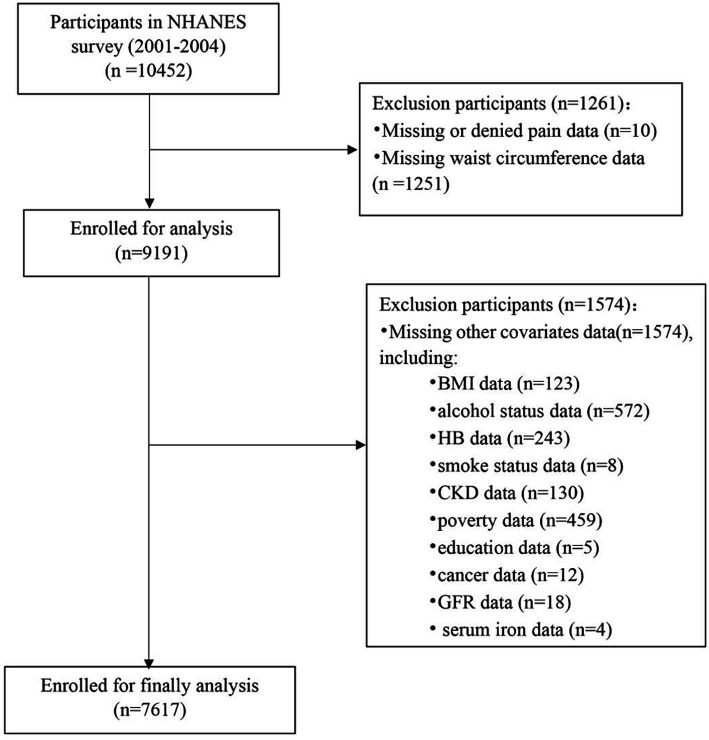
Flow chart of the study population.

The NHANES includes interviews and physical exams. The survey consists of five main parts: demographic data, laboratory tests, dietary information, physical exams, and questionnaire responses. Our examination was limited to individuals who were at least 20 years old and older from the NHANES datasets between 2001 and 2004, and who had filled out the Miscellaneous Pain Questionnaire. The final analysis included 7,617 participants out of 10,452 subjects surveyed between 2001 and 2004, following the application of exclusion criteria (Figure 1). Exclusion criteria involved missing or denied pain data (*n* = 10), missing waist circumference data (*n* = 1,251), and other missing covariate data (*n* = 1,574); missed BMI data (*n* = 123), missed alcohol status (*n* = 572), missed HB data (*n* = 243), missed smoke status (*n* = 8), missed CKD data (*n* = 130), missed poverty data (*n* = 459), missed education data (*n* = 5), missed cancer data (*n* = 12), missed GFR data (*n* = 18), missed serum iron data (*n* = 4).

#### 2.1.2 Variables

Waist circumference (dm) was the primary independent variable examined in the research, while chronic pain was served as the dependent variable. The study assessed chronic pain by examining two factors: MPQ100, which signifies pain lasting over 24 h in the previous month, and MPQ110, which measures the duration of the pain. Chronic pain was described as pain that continues or repeats for over 3 months, and participants with chronic pain were identified by having pain issues for 3 months or longer (MPQ100 = 1, MPQ110 = 3 or 4) (25, 26).

Covariates were classified into three groups: demographic data, medical conditions, and examination results. Demographic factors such as age, gender, education, poverty-income ratio (PIR), smoke status (never: smoked less than 100 cigarettes in life; former: smoked more than 100 cigarettes in life and smoke not at all now; now: smoked more than 100 cigarettes in life and smoked some days or every day), alcohol status (never: had <12 drinks in lifetime; former: had ≥12 drinks in 1 year and did not drink last year, or did not drink last year but drank ≥12 drinks in lifetime; mild: 1 drink per day is for female and 2 drinks per day is for male; moderate: 2 drinks per day is for female and 3 drinks per day is for male, or binge drinks ≥2 days but <5 days per month; heavy: 3 drinks per day is for female and 4 drinks per day is for male, or binge drinks ≥5 days per month) (27), and obesity (body mass index (BMI) < 30 kg/m^2^; BMI ≥ 30 kg/m^2^)were considered. Medical conditions like chronic kidney disease (CKD), cardiovascular disease (CVD), hyperlipidemia, cancer, anemia were also taken into account. Examination results were consisted of albumin, alanine aminotransferase (ALT), white blood cell (WBC), hemoglobin (HB), glomerular filtration rate (GFR), red cell distribution width (RWD).

### 2.2 Mendelian randomization

It is widely acknowledged that cross-sectional studies, due to their inherent design, cannot control for all confounding factors, leading to potential biases in results. MR analysis, in contrast, offers a method to circumvent such limitations by examining the relationship between waist circumference and chronic pain from a genetic epidemiological perspective, thus enabling the establishment of a potential causal link. MR utilizes instrumental variables, such as single nucleotide polymorphisms (SNPs), for assessing causality between exposure and outcome (28). SNPs, as the most frequent type of genetic variation, occur when there’s a switch of a single nucleotide—adenine (A), thymine (T), cytosine (C), or guanine (G)—along the DNA sequence. They act as surrogates for exposure variables, randomly allocated at birth, which minimizes the impact of external factors and lifestyle choices on the analysis. And genome-wide association studies (GWAS) enable the aggregation of genetic effects from numerous SNPs linked to specific traits, thereby estimating the proportion of trait variability that can be attributed to these SNPs (29). This approach is instrumental in pinpointing individuals at elevated risk who might benefit from specific preventive measures. So this MR analysis, less affected by environmental confounders and reverse causality than traditional observational studies, was considered nearly as robust as randomized controlled trials, but was more cost-effective, time-efficient, and faced fewer ethical constraints (28, 30–32).

Genetic data for pooled waist circumference and pain levels were obtained from publicly available sources. In order to address potential bias caused by population admixture, the population genetic background of the MR study was restricted to individuals of European ancestry. This study (GWAS ID ukb-b-9405) included 462,166 participants of European descent and identified genome-wide significant SNPs (*p* < 5 × 10^−8^) as independent predictors of waist circumference (r^2^ < 0.001, kb = 10,000). The genetic information related to pain was obtained from the ninth edition of the Finngen Biobank. This dataset consisted of 171,922 individuals diagnosed with pain and 204,598 individuals without pain (controls). We utilized approximately 20.1 million SNPs for the association analyses. The pain patients were identified using ICD10 and ICD9 diagnosis codes, which included limb, back, neck, and abdominal pain (more information on the above can be found at https://r9.risteys.finngen.fi/endpoints/PAIN).

Additionally, given that waist circumference differed between female and male sexes, we used gender-specific IEU GWAS data of waist circumference to explore whether the relationship between waist circumference and pain was influenced by sex. These two GWAS data sets had 104,079 male (Male GWAS ID: ieu-a-71) and 127,469 female (Female GWAS ID: ieu-a-69) participants of European, respectively.

Furthermore, repeated MR analyses were performed using other three waist circumference GWAS datasets from IEU GWAS, followed by a meta-analysis to consolidate causal relationship between waist circumference and pain. These three GWAS data sets included 407,661 (GWAS id: ebi-a-GCST90014020), 336,639 (GWAS id: ukb-a-382), and 232,101 (GWAS id: ieu-a-61) participants of European, respectively.

All GWAS studies included in this research were approved by the relevant ethical review boards, and participants provided written informed consent. The study was carried out following the STROBE MR guideline (33). The utilization of solely accessible summary-level data obviated the necessity for supplementary ethical assessment.

## 3 Statistical analysis

### 3.1 NHANES

Our study took into account intricate sampling methodologies and weights, using mobile examination center (MEC) weights for all analyses. Baseline characteristics were expressed as means and standard errors (SE) for continuous variables and as proportions for categorical variables. WC was categorized into quartiles. WC quartile groups were compared using Student’s t-tests for continuous variables and chi-square tests for categorical variables. A logistic regression model was used to calculate the odds ratio (OR) and 95% confidence intervals (CI) to examine the relationship between WC and chronic pain.

Our statistical inferences were based on three models: crude model had no adjusted variables. Model 1 incorporated age, sex, education, and poverty income ratio (PIR). Model 2 included all variables from Model 1, in addition to hemoglobin (HB), white blood cell (WBC), chronic kidney disease (CKD), glomerular filtration rate (GFR), red cell distribution width (RWD), alcohol status, smoke status, obesity, albumin, anemia, Hyperlipidemia, cancer.

This study stratified users based on age (individuals over 60, individuals under 60), sex (male, female). An analysis of stratification explored the correlation between WC and chronic pain. The restricted cubic splines (RCS) regression model was used to flexibly analyze the relationship between WC and chronic pain. R Studio 4.2.0 was utilized for conducting statistical analyses. A significant level of *p* < 0.05 was established.

### 3.2 Primary analysis of MR

Regarding the MR analyses, we computed F statistics to assess the potency of every instrument. Inverse variance weighted (IVW) was the primary method for assessing the association between genetically predicted waist circumference and pain risk. We also used MR-Egger and weighted mode (WM) to validate the results from IVW. Prior publications had examined the pros and cons of these approaches (34, 35). To assess potential heterogeneity and directional pleiotropy, we employed the Cochrane Q test and MR-Egger intercept separately (35). Sensitivity analysis with leave-one-out method, forest plot, and funnel plot were also conducted. Furthermore, we used the Phenoscanner V2 website^1^ to explore whether the genetic variants associated with waist circumference were also connected to other prevalent risk factors that might affect the results obtained from Mendelian randomization, including poor mental status or psychiatric disorders (nerves, anxiety, tension, depression or schizophrenia) (36, 37), diabetes (38), coronary artery disease (39), gout (40), and alcohol (41). Once the association of the SNPs and these potential confounders reached the threshold of *p* < 1 × 10^−5^, we replicated the instrumental variable analysis using the IVW method after dropping the associated SNPs to ensure the reliability and credibility of genetic epidemiology studies.

### 3.3 Stratification analyses, validation, and meta-analysis of MR

To determine whether the relationship between waist circumference and pain is influenced by sex, we performed a stratified analysis using gender-specific GWAS waist circumference data. And to further validate the reliability of the MR analysis results, we utilized external cohorts for verification. For the validation process and stratification analyses, we performed the analysis using the same methods as described in the original MR analysis. The analyzed results were then subjected to a meta-analysis to combine the effect sizes. The I^2^ statistic was employed to assess the heterogeneity of the meta-analysis. When I^2^ ≤ 50% and the *p*-value ≥ 0.05, a fixed-effects model was adopted. For cases where I^2^ > 50% and the *p*-value < 0.05, the random-effects model was utilized. The Review Manager software (Version 5.3) was utilized for conducting statistical analyses.

## 4 Results

### 4.1 NHANES

#### 4.1.1 Baseline characteristics

The data analyzed was sourced from 7,617 participants. WC was divided into quartiles: Q1 (<8.70 decimetre), Q2 (8.70–9.68 decimetre), Q3 (9.68–10.67 decimetre), Q4 (≥ 10.67 decimetre). The baseline characteristics were displayed in Table 1 based on the WC quartiles. The analysis showed that participants with higher WC levels were typically older males, CKD, CVD, smoking status. They also had lower serum iron, and albumin concentrations. They also had higher HB, RDW, Alt, and WBC (Table 1, all *p* < 0.05).

**Table 1:** Baseline characteristics of study participants based on the WC quartile.

Variables	Total (*n* = 7,617)	Q1 (<8.70 dm; *n* = 1907)	Q2 (8.70–9.68 dm; *n* = 1935)	Q3 (9.68–10.67 dm; *n* = 1878)	Q4 (≥10.67 dm; *n* = 1897)	*p*-value
Age, (yr)	45.657 (0.366)	40.260 (0.429)	45.427 (0.663)	49.222 (0.426)	48.910 (0.416)	<0.001
Iron, (ug/dl)	89.433 (0.581)	94.990 (1.051)	90.503 (1.333)	89.089 (1.049)	82.005 (0.820)	<0.001
HB, (g/dl)	14.527 (0.058)	14.172 (0.070)	14.618 (0.076)	14.696 (0.067)	14.691 (0.073)	<0.001
WBC, (×10^9^/L)	7.261 (0.042)	6.960 (0.050)	7.154 (0.076)	7.311 (0.080)	7.685 (0.090)	<0.001
eGFR, (ml/min)	94.348 (0.570)	99.006 (0.724)	94.552 (0.925)	91.047 (0.679)	91.753 (0.550)	<0.001
RWD, (%)	12.623 (0.019)	12.487 (0.030)	12.564 (0.032)	12.623 (0.022)	12.848 (0.030)	<0.001
ALT, (U/L)	26.071 (0.424)	22.024 (1.274)	25.276 (0.565)	27.628 (0.448)	30.240 (0.668)	<0.001
Albumin, (g/L)	42.720 (0.075)	43.494 (0.085)	42.965 (0.096)	42.673 (0.099)	41.583 (0.100)	<0.001
Sex, *n* (%)						<0.001
Female	3,897 (51.029)	1,228 (68.960)	989 (49.274)	827 (40.724)	853 (41.400)	
Male	3,720 (48.971)	679 (31.040)	946 (50.726)	1,051 (59.276)	1,044 (58.600)	
Education, *n* (%)						<0.001
9-11th Grade	1,135 (11.314)	271 (11.094)	300 (11.721)	263 (10.758)	301 (11.680)	
College Graduate or above	1,556 (25.858)	485 (31.852)	390 (24.647)	327 (22.298)	354 (23.410)	
High School Grad/GED or Equivalent	1,830 (25.985)	407 (21.760)	468 (27.004)	473 (27.340)	482 (28.647)	
Less Than 9th Grade	1,007 (5.902)	192 (4.800)	261 (6.024)	309 (7.367)	245 (5.680)	
Some College or AA degree	2,089 (30.941)	552 (30.494)	516 (30.603)	506 (32.237)	515 (30.584)	
Cancer, *n* (%)						<0.001
No	6,931 (91.522)	1,782 (93.701)	1,768 (92.556)	1,682 (90.100)	1,699 (89.199)	
Yes	686 (8.478)	125 (6.299)	167 (7.444)	196 (9.900)	198 (10.801)	
Anemia, *n* (%)						0.330
No	7,107 (95.538)	1,782 (94.926)	1,820 (96.202)	1,755 (95.779)	1,750 (95.335)	
Yes	510 (4.462)	125 (5.074)	115 (3.798)	123 (4.221)	147 (4.665)	
Hyperlipidemia, *n* (%)						<0.001
No	1965 (28.215)	867 (47.697)	498 (28.446)	302 (16.613)	298 (15.887)	
Yes	5,652 (71.785)	1,040 (52.303)	1,437 (71.554)	1,576 (83.387)	1,599 (84.113)	
CVD, n (%)						<0.001
No	6,773 (91.711)	1,799 (96.034)	1752 (92.993)	1,618 (89.140)	1,604 (87.733)	
Yes	843 (8.274)	107 (3.966)	183 (7.007)	260 (10.860)	293 (12.267)	
CKD, *n* (%)						<0.001
No	6,252 (87.184)	1,672 (90.729)	1,610 (88.774)	1,513 (86.096)	1,457 (82.325)	
Yes	1,365 (12.816)	235 (9.271)	325 (11.226)	365 (13.904)	440 (17.675)	
Alcohol status^#^, *n* (%)						<0.001
Former	1,567 (17.195)	272 (10.831)	374 (16.233)	424 (18.613)	497 (24.437)	
Heavy	1,452 (20.711)	397 (21.955)	388 (21.592)	347 (20.963)	320 (18.054)	
Mild	2,469 (34.240)	621 (33.626)	622 (34.616)	644 (37.016)	582 (31.905)	
Moderate	1,049 (15.804)	347 (20.888)	277 (16.237)	195 (11.409)	230 (13.510)	
Never	1,080 (12.051)	270 (12.700)	274 (11.323)	268 (11.999)	268 (12.093)	
Smoke status^&^, *n* (%)						<0.001
Former	2065 (25.357)	365 (19.015)	492 (24.370)	570 (27.671)	638 (31.736)	
Never	3,844 (49.650)	1,009 (52.158)	1,019 (50.364)	936 (50.270)	880 (45.311)	
Now	1708 (24.993)	533 (28.827)	424 (25.266)	372 (22.059)	379 (22.952)	
PIR, *n* (%)						0.098
<1.5	2,410 (22.940)	600 (24.246)	605 (22.678)	583 (20.560)	622 (23.947)	
≥1.5	5,207 (77.060)	1,307 (75.754)	1,330 (77.322)	1,295 (79.440)	1,275 (76.053)	
Obesity, *n* (%)						<0.001
BMI < 30 kg/m^2^	5,188 (69.122)	1898 (99.624)	1780 (91.519)	1,237 (65.829)	273 (12.292)	
BMI ≥30 kg/m^2^	2,429 (30.878)	9 (0.376)	155 (8.481)	641 (34.171)	1,624 (87.708)	
Chronic pain, *n* (%)						<0.001
No	6,448 (83.122)	1,668 (86.398)	1,678 (84.734)	1,563 (80.468)	1,539 (80.065)	
Yes	1,169 (16.878)	239 (13.602)	257 (15.266)	315 (19.532)	358 (19.935)	

Data were presented as mean and standard errors (SE) for continuous variables, number and proportions for categorical variables. dm, decimeter; HB, hemoglobin; WBC, white blood cell; GFR, glomerular filtration rate; RWD, red cell distribution width; ALT, alanine aminotransferase; CVD, cardiovascular disease; CKD, Chronic kidney disease; PIR, poverty income ratio; BMI, body mass index.

# Alcohol status (never: had < 12 drinks in lifetime; former: had ≥ 12 drinks in 1 year and did not drink last year, or did not drink last year but drank ≥ 12 drinks in lifetime; mild: 1 drink per day is for female and 2 drinks per day is for male; moderate: 2 drinks per day is for female and 3 drinks per day is for male, or binge drinks ≥ 2 days but < 5 days per month; heavy: 3 drinks per day is for female and 4 drinks per day is for male, or binge drinks ≥ 5 days per month).

& Smoke status (never: smoked less than 100 cigarettes in life; former: smoked more than 100 cigarettes in life and smoke not at all now; now: smoked more than 100 cigarettes in life and smoked some days or every day).

#### 4.1.2 Association between WC and chronic pain

The WC was analyzed both as a continuous and a categorized variable (four groups) in Table 2. The continuous model (per 1 decimetre) revealed a strong link between WC and chronic pain across all models following adjustments for multiple variables, as for crude model (OR = 1.13, 95%CI: 1.07–1.18 *p* < 0.001), Model 1 (OR = 1.13, 95%CI: 1.07–1.19 p < 0.001), Model 2 (OR = 1.14, 95%CI: 1.04–1.24, *p* = 0.01).

**Table 2:** The association between waist circumference and chronic pain.

	Crude model		Model 1		Model 2	
	OR (95%CI)	*p*-value	OR (95%CI)	*p*-value	OR (95%CI)	*p*-value
**Continuous**						
WC (per 1 dm)	1.13 (1.07,1.18)	<0.001	1.13 (1.07,1.19)	<0.001	1.14 (1.04,1.24)	0.010
**Categorized**						
Q1 (<8.70 dm)	Reference		Reference		Reference	
Q2 (8.70–9.68 dm)	1.14 (0.90,1.46)	0.260	1.17 (0.93,1.46)	0.160	1.20 (0.91,1.59)	0.160
Q3 (9.68–10.67 dm)	1.54 (1.24,1.92)	<0.001	1.62 (1.30,2.02)	<0.001	1.68 (1.22,2.32)	0.010
Q4 (≥ 10.67 dm)	1.58 (1.28,1.95)	<0.001	1.63 (1.29,2.05)	<0.001	1.64 (1.07,2.52)	0.030
*p* for trend		<0.001		<0.001		0.010

Crude model: Unadjusted; Model 1: Adjusted for age, sex, education, poverty income ratio (PIR); Model 2: Adjusted for age, sex, education, poverty income ratio (PIR), hemoglobin (HB); white blood cell (WBC), chronic kidney disease (CKD), glomerular filtration rate (GFR), red cell distribution width (RWD), alcohol status, smoke status, obesity, albumin, anemia, hyperlipidemia, cancer. OR, odds ratio; CI, confidence intervals; dm, decimeter.

As a categorized variable, in order to mitigate the impact of outliers on the analysis. After adjusting for multiple variables (model 2), the top quartile of WC was still significantly associated with chronic pain (OR = 1.64, 95% CI: 1.07–2.52, *p* = 0.030) when compared to the lowest quartile of WC. The association between WC and chronic pain is detailed in Table 2.

**Figure 2:**
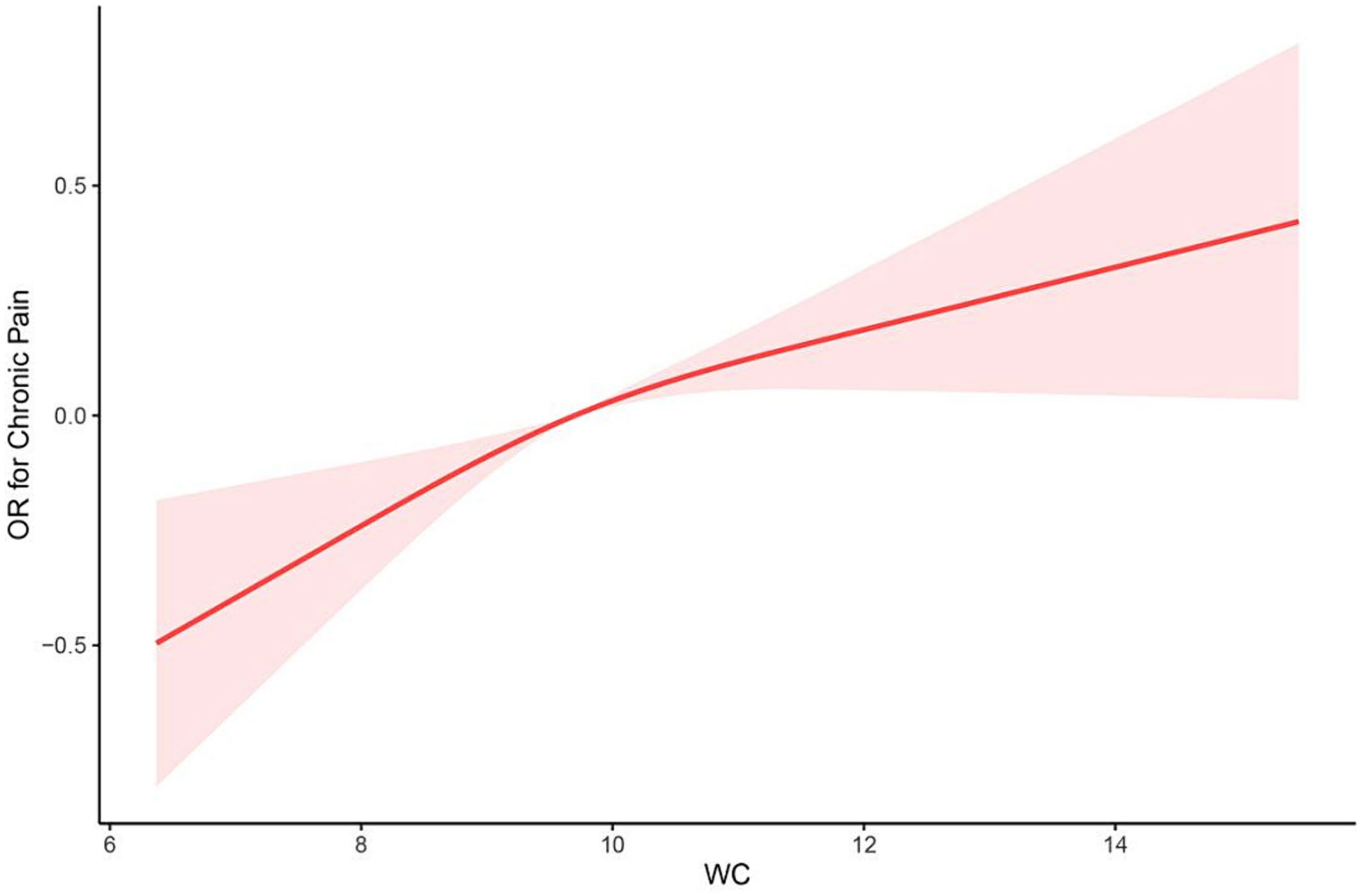
The restricted cubic spline (RCS) curve between WC and chronic pain among participants in NHANES 2001–2004 (*p* = 0.267 for nonlinearity). OR, odds ratio; WC, waist circumference.

After multivariate adjustments (model 2), the RCS model found that the relationship between the WC and chronic pain presented a linear (*p* = 0.267 for nonlinearity; Figure 2).

#### 4.1.3 Stratification analyses

Subgroup analysis based on age, as well as sex, revealed that the impact of waist circumference on chronic pain was consistent in different population (all *p* < 0.05). The stratification analyses further supported the robustness of the results in different population (both *p* for interaction >0.05, Table 3).

**Table 3:** Results of stratification analyses.

Variables	OR (95% CI)	*p*-value	*p* for interaction
Aged			0.673
<60	1.078 (1.009,1.153)	0.030	
≥60	1.149 (1.050,1.257)	0.005	
Sex			0.821
Male	1.101 (1.013,1.197)	0.026	
Female	1.122 (1.033,1.218)	0.009	

CI, confidence intervals.

### 4.2 MR analyses using primary genetic instruments

#### 4.2.1 Primary outcome of MR analysis between waist circumference and pain

**Figure 3:**
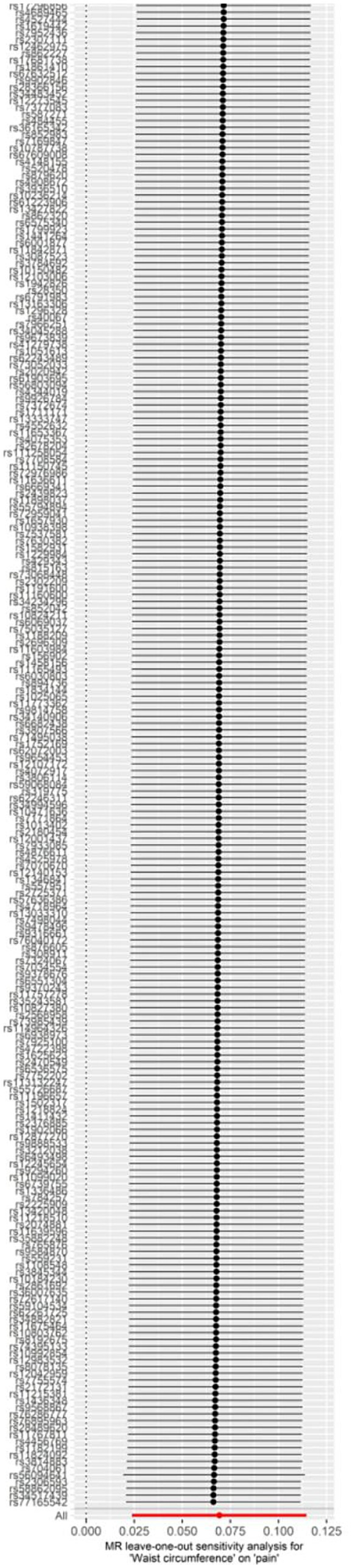
Leave-one-out plot for the significant Mendelian randomization (MR) association between waist circumference and pain.

At first, 234 SNPs were utilized in the genetic instrument for waist circumference. According to funnel and forest plot, we removed one SNP (rs156902) because it had heterogeneity with other SNPs (Supplementary Figures S2, S3). Finally, 233 SNPs were used for final analysis (Supplementary Table S1). The Cochrane Q test revealed no significant heterogeneity (*p* = 0.263), and the MR-Egger regression did not provide evidence for horizontal pleiotropy (*p* = 0.246). The results showed that waist circumference was linked to a greater likelihood of experiencing pain (OR = 1.072, 95% CI: 1.024–1.121, *p* = 0.003) using the IVW method. The result of the MR Egger method (OR = 1.001, 95% CI: 0.886–1.132, *p* = 0.981) and weighted mode (WM; OR = 1.036, 95% CI: 0.914–1.176, *p* = 0.580) were not statistically significant (Supplementary Figure S1). The leave-one-out analysis revealed no significant effect of any single SNP that might dominate the results (Figure 3).

The results of the analysis found no evidence of bias that would invalidate the estimates. However, we recognized the importance of further investigation into the second traits associated with the waist circumference SNPs. Therefore, we conducted a manual investigation into the traits of poor mental status or psychiatric disorders (nerves, anxiety, tension, depression or schizophrenia), diabetes, coronary artery disease, gout, and alcohol. After reviewing the Phenoscanner, we discovered that 62 SNPs were linked to these diseases. Then we removed these 62 SNPs and found that the causality remained the same (IVW OR = 1.074, 95% CI: 1.015–1.137, *p* = 0.014).

#### 4.2.2 Stratification analyses, validation and meta-analysis of MR between waist circumference and pain

The stratification analyses of MR showed that there was no causal relationship between waist circumference and pain both in male (OR = 1.039, 95%CI: 0.941–1.148, *p* = 0.447) or female (OR = 0.938, 95%CI: 0.860–1.024, *p* = 0.154) participants.

**Figure 4:**
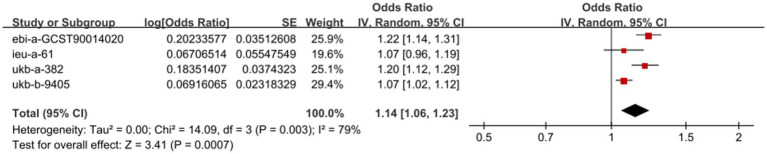
Meta-analysis of MR on the relationship between waist circumference and pain.

This study used three other GWAS data of waist circumference to repeat the analysis of the causal relationship between waist circumference and pain. Two of them showed that waist circumference had a causal relationship with pain (GWAS ID: ebi-a-GCST90014020, OR = 1.224, 95%CI: 1.143–1.312, *p* = 8.397 × 10^−9^; GWAS ID: ukb-a-382, OR = 1.201, 95%CI: 1.116–1.293, *p* = 9.460 × 10^−7^), while another GWAS data found that there was no causal association between waist circumference and pain (GWAS ID: ieu-a-61, OR = 1.069, 95%CI: 0.959–1.192, *p* = 0.227). However, the meta-analysis of MR showed increased waist circumference with a genetic predisposition for pain risk (OR = 1.14, 95%CI: 1.06–1.23, *p* = 0.0007; Figure 4).

## 5 Discussion

In this investigation, our analysis of the nationally representative NHANES 2001–2004 cross-sectional data indicated a significant finding: 1 dm increase in waist circumference was associated with a 14% increase in chronic pain prevalence. Furthermore, our meta-analysis of MR suggested that waist circumference had a causal relationship with pain.

Researches focusing on waist circumference as an obesity metric in relation to chronic pain are currently limited and yield varied conclusions. For instance, Sultana Monira Hussain’s study (42), which encompassed a 12-year Australian cohort of 5,058 individuals, identified a positive correlation between waist circumference and chronic lower back pain after adjustment for confounders. Stella Muthuri’s research (43) in a 32-year UK birth cohort of 3,426 participants also found higher waist circumference to be linked with a greater likelihood of adult-onset back pain. A comprehensive meta-analysis (44) encompassing 10 studies related to waist circumference concluded that a higher waist circumference, irrespective of BMI status, increases the risk of chronic lower back pain and is also associated with other chronic pain types. These findings align with our analysis of a substantial NHANES chronic pain sample, but we also provided causal validation through MR analysis and improved the exclusion of confounding factors. Furthermore, we conducted gender-specific subgroup analyses in the cross-sectional study, revealing a positive correlation between waist circumference and chronic pain in both males and females. This finding contrasts with Qiqi You’s meta-analysis (44), which found no correlation for men’s waist circumference with lower back pain risk, but noted a positive association for women. However, we conducted a stratified MR analysis and found no influence of gender on the relationship between waist circumference and pain, both male and female waist circumferences were not related to pain. This finding is inconsistent with the results of the subsequent meta-analysis we conducted. We speculate that this inconsistency may be due to the limitations in the number of participants and SNPs in the existing GWAS waist circumference databases. In summary, the impact of gender on the relationship between waist circumference and chronic pain requires further research.

Recently, MR analysis was commonly used to explore a causal relationship. Previous studies using this approach found that waist circumference had a causal association with sciatica, low back pain, knee pain, and hip pain (45–48). In our study, we also further validated that the causal relationship between waist circumference and pain using other three waist circumference GWAS datasets from IEU GWAS, followed by a meta-analysis of MR. Furthermore, our study included all types of pain, and the results were supported by cross-sectional data, which provides greater confidence.

The underlying mechanisms of increased waist circumference leading to chronic pain may be multifactorial. Elevated waist circumference indicates abdominal fat accumulation, linked to alterations in non-esterified fatty acid (NEFA) metabolism and endocrine dysfunction (49), exacerbating gravitational load on the spine and potentially altering lower back intervertebral disc structure (50, 51), possibly contributing to chronic lower back pain. Moreover, adipose tissue, being metabolically active, can induce systemic inflammation through pro-inflammatory molecules such as tumor necrosis factor, leptin, and interleukin (52). Chronic inflammation can lead to atherosclerosis (53) and is associated with central and peripheral sensitization of pain perception (54), providing potential explanations for why waist circumference can lead to chronic pain throughout the body.

Our study’s strengths include leveraging a large cross-sectional sample and validating the unidirectional causality between waist circumference and pain using two-sample MR analysis, which is less prone to reverse causality and confounding, thereby enhancing result credibility. This novel approach has not been previously employed to investigate the waist circumference-pain relationship. However, our research is limited by the cross-sectional analysis, which did not account for dietary factors due to data limitations, potentially leading to bias. Additionally, while NHANES provides exposure at a specific time point, MR assesses lifelong effects. Although the MR Egger intercept did not indicate horizontal pleiotropy, this possibility cannot be completely ruled out. Furthermore, our study discovered a linear correlation between waist circumference and chronic pain, but database constraints prevented examination of a linear causal relationship in the MR analysis. Moreover, the use of chronic pain data in the cross-sectional study but only pain data in the MR analysis may also introduce bias. Considering the extraordinarily complex factors that influence chronic pain, it is challenging to address all confounding factors, which inevitably limits the scope of our findings. Finally, the diversity of the databases, encompassing various U.S. ethnic groups in NHANES and primarily European populations for genetic data (waist circumference from Europeans, and pain data from Northern Europeans), may limit the generalizability of the results. Future studies should aim to replicate similar research within more homogenous ethnic groups to mitigate these limitations.

## 6 Conclusion

In summary, observational analysis confirmed a significant relationship between increased waist circumference and the incidence of chronic pain, and results from MR Study identified increased waist circumference as potentially causal for pain. Based on this insight, it is reasonable for advocating waist circumference control in the management strategies for chronic pain.

## Data availability statement

The original contributions presented in the study are included in the article/Supplementary material, further inquiries can be directed to the corresponding authors.

## Ethics statement

The studies involving humans were approved by NCHS Research Ethics Review Board. The studies were conducted in accordance with the local legislation and institutional requirements. The human samples used in this study were acquired from gifted from another research group. Written informed consent for participation was not required from the participants or the participants’ legal guardians/next of kin in accordance with the national legislation and institutional requirements.

## Author contributions

TX: Data curation, Formal analysis, Investigation, Methodology, Software, Writing – original draft, Writing – review & editing. FJ: Data curation, Formal analysis, Software, Writing – original draft, Writing – review & editing. YY: Data curation, Formal analysis, Software, Writing – review & editing. JH: Data curation, Investigation, Writing – review & editing. RY: Formal analysis, Investigation, Writing – review & editing. TL: Investigation, Methodology, Project administration, Supervision, Validation, Writing – review & editing. ZY: Conceptualization, Investigation, Methodology, Project administration, Resources, Supervision, Validation, Writing – review & editing.

## Footnotes

^1^ http://www.phenoscanner.medschl.cam.ac.uk/
