# Supplementary figures and images for: Association between waist circumference and chronic pain: insights from observational study and two-sample Mendelian randomization
Source: Front Nutr. 2024 Jul 26;11:1415208. doi: 10.3389/fnut.2024.1415208 (PMC11310123; doi:10.3389/fnut.2024.1415208)

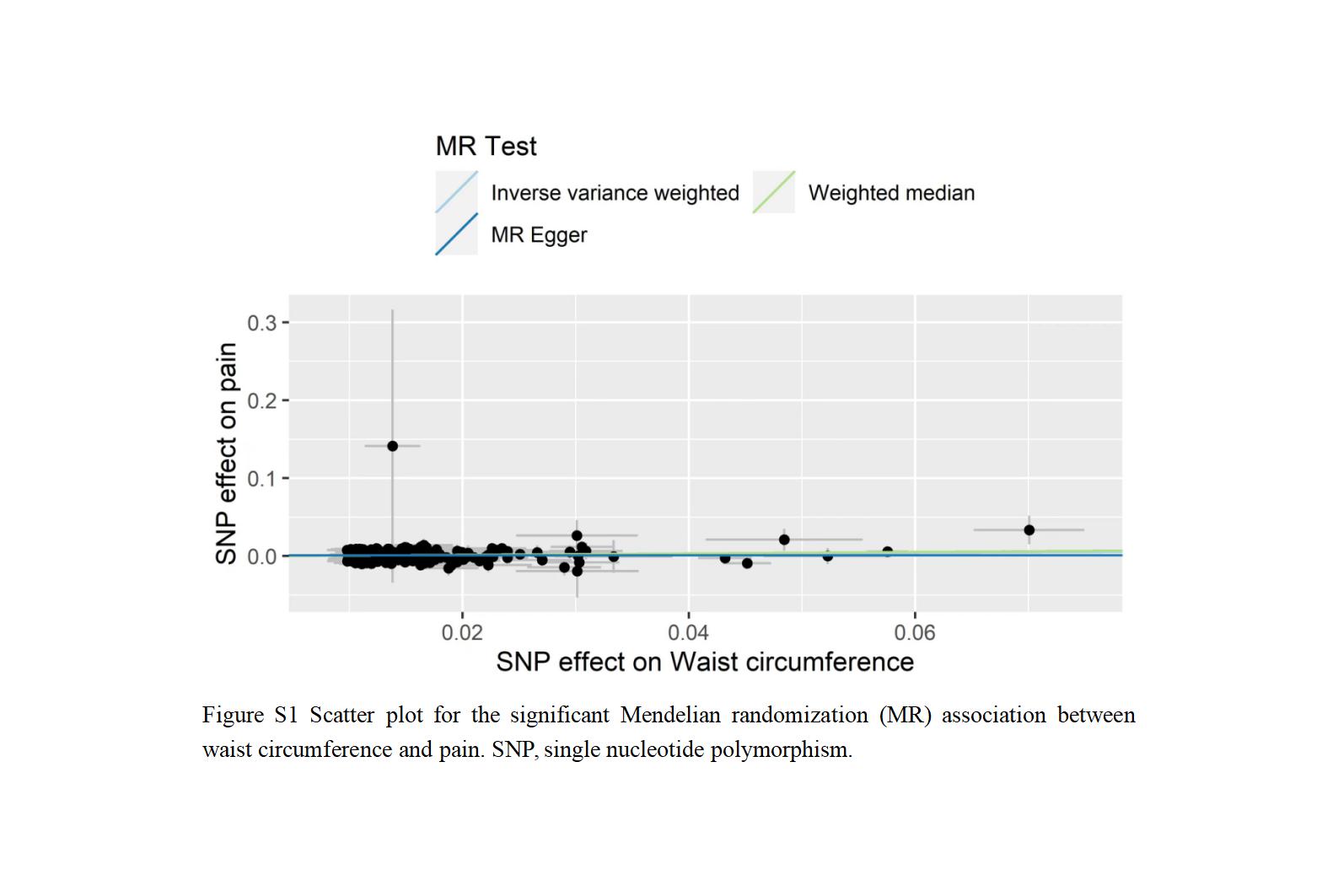

Supplement: Supplementary file 2 [file Image_1.JPEG]

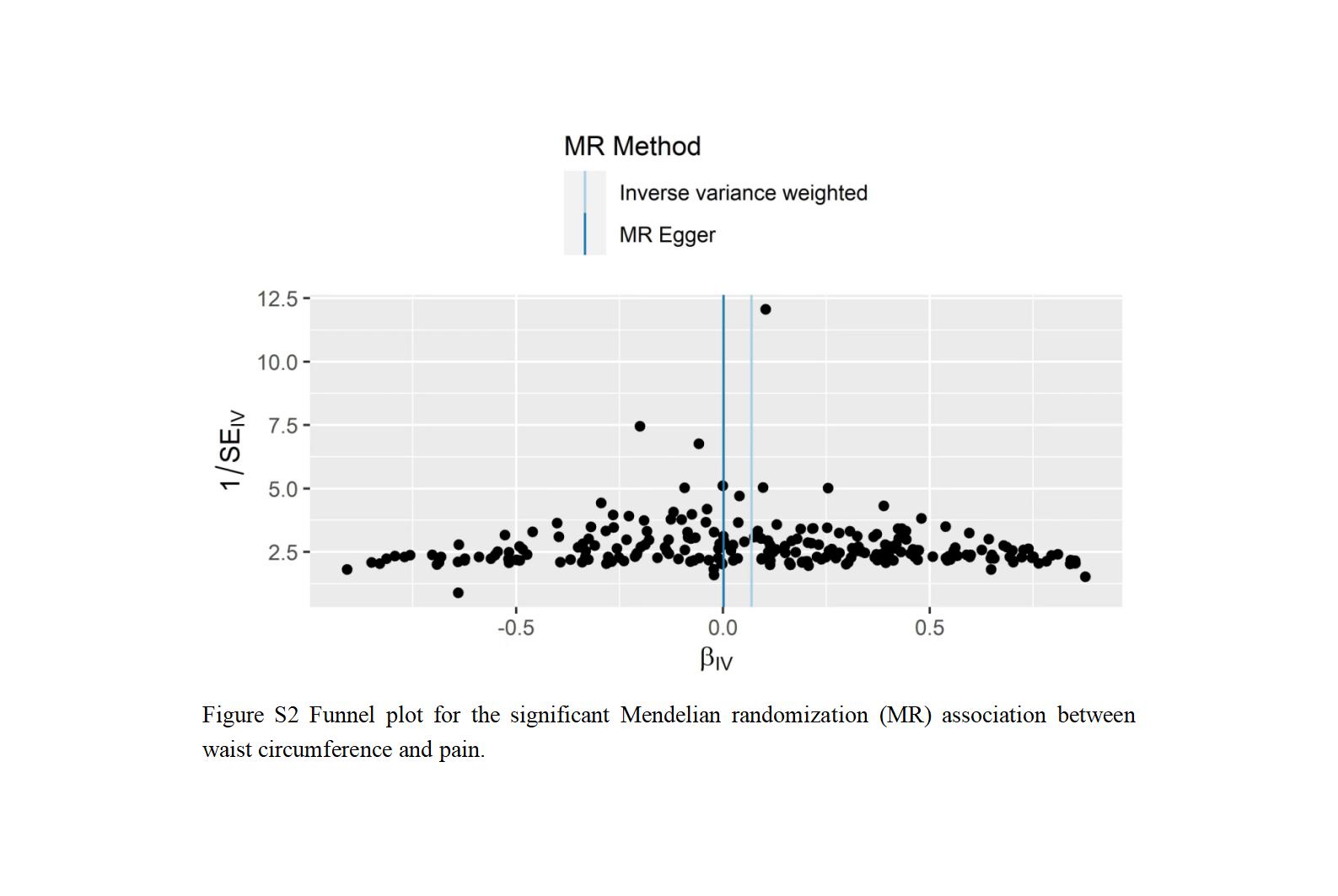

Supplement: Supplementary file 3 [file Image_2.JPEG]

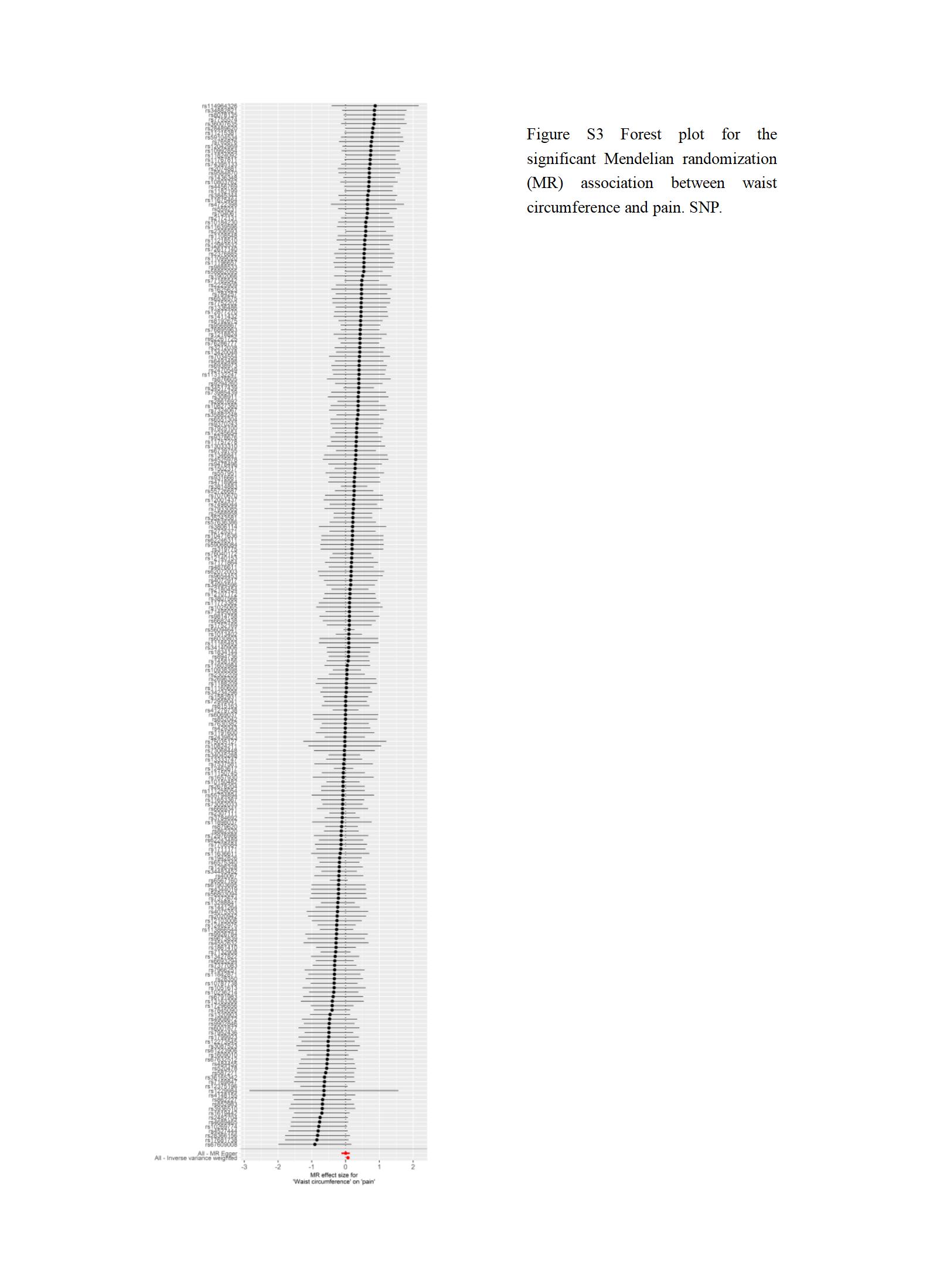

Supplement: Supplementary file 4 [file Image_3.JPEG]
